# Heterozygous Alterations of *TNFRSF13B/TACI* in Tonsillar Hypertrophy and Sarcoidosis

**DOI:** 10.1155/2013/532437

**Published:** 2013-07-15

**Authors:** Matthaios Speletas, Ulrich Salzer, Zoe Florou, Efthimia Petinaki, Zoe Daniil, Fotini Bardaka, Konstantinos I. Gourgoulianis, Charalampos Skoulakis, Anastasios E. Germenis

**Affiliations:** ^1^Department of Immunology and Histocompatibility, Faculty of Medicine, School of Health Sciences, University of Thessaly, Biopolis, 41110 Larissa, Greece; ^2^Centre of Chronic Immunodeficiency (CCI), University Medical Center Freiburg and University of Freiburg, Hugstetterstrabe 55, 79106 Freiburg, Germany; ^3^Department of Microbiology, Faculty of Medicine, School of Health Sciences, University of Thessaly, Biopolis, 41110 Larissa, Greece; ^4^Respiratory Department, University Hospital of Larissa, Faculty of Medicine, School of Health Sciences, University of Thessaly, Biopolis, 41110 Larissa, Greece; ^5^ENT Department, University Hospital of Larissa, Faculty of Medicine, School of Health Sciences, University of Thessaly, Biopolis, 41110 Larissa, Greece

## Abstract

*TNFRSF13B/TACI* defects have been associated with CVID pathogenesis and/or phenotype, especially the development of benign lymphoproliferation and autoimmunity. Our purpose was to investigate the role of *TNFRSF13B/TACI* defects in the pathogenesis of two common lymphoproliferative disorders, namely, sarcoidosis and tonsillar hypertrophy (TH). 105 patients (71 with sarcoidosis and 34 with TH, including 19 without infectious causative and 15 due to *Haemophilus influenzae*) were analyzed for *TNFRSF13B/TACI* defects. Two out of 19 TH patients without infectious cause (10.5%) and 2 patients with sarcoidosis (2.8%) displayed rare *TNFRSF13B/TACI* defects (I87N, L69TfsX12, E36L, and R202H, resp.). Both mutations identified in TH patients have been assessed as deleterious for protein function, while the patient with the R202H mutation and sarcoidosis exhibited also sIgG4D. Our study further supports the notion that *TNFRSF13B/TACI* defects alone do not result in CVID but may be also found frequently in distinct clinical phenotypes, including benign lymphoproliferation and IgG subclass deficiencies.

## 1. Introduction

Recent studies have demonstrated that *TNFRSF13B/TACI* mutations may contribute to the pathogenesis and/or phenotype of CVID [1–4]. Although the first of them have ascribed a pathogenic role of *TNFRSF13B/TACI* alterations in CVID [1, 2], further studies suggested that these mutations might represent modifiers of the disease, since they are also present in healthy individuals, without any evidence of immunodeficiency [3, 4]. However, *ex vivo* and animal studies have indicated that some of these mutations have a profound effect on B-cell function, even in the heterozygous state [5, 6]. Moreover, Salzer et al. identified that the presence of specific *TNFRSF13B/TACI* mutations, as C104R and A181E, has been associated with both a high risk of CVID development and specific aspects of disease phenotype, that is, the presence of benign lymphoproliferation, such as splenomegaly, lymphadenopathy, and autoimmune complications [3].

CVID patients exhibit an increased incidence of autoimmunity and lymphoproliferation [7, 8], while approximately 10% of them display granulomatous inflammatory reactions, which can be accompanied by interstitial lymphoid hyperplasia in lungs (GLILD), a pathologic condition clinically indistinguishable from sarcoidosis [9]. It is worth mentioning that *TNFRSF13B/TACI* knockout mice develop lymphoproliferation, lupus-like nephritis, and elevated levels of circulating autoantibodies [10, 11]. These findings have raised the question whether *TNFRSF13B/TACI* mutations contribute to the pathogenesis of human pathologic conditions similar to those observed in TACI-deficient mice, or diseases with clinical manifestations similar to those observed in CVID, such as sarcoidosis. Until now, only one study has analyzed the role of *TNFRSF13B/TACI* in systemic lupus erythematosus but did not reveal any deleterious or disease-associated mutations into the coding region of the gene [12]. However, the contribution of *TNFRSF13B/TACI* alterations in the pathogenesis of human lymphoproliferative disorders, malignant or benign, is elusive. 

The aim of this study was to investigate the role of *TNFRSF13B/TACI* alterations in the pathogenesis of two common benign lymphoproliferative disorders, namely, sarcoidosis and tonsillar hypertrophy. Furthermore, we analyzed whether the presence of *TNFRSF13B/TACI* alterations in a large cohort of CVID patients also affected the risk of tonsillar hypertrophy. 

## 2. Material and Methods

### 2.1. Patients

One hundred and five (105) patients were enrolled in the study. Among them, 71 patients suffered from sarcoidosis, 19 from tonsillar hypertrophy (TH) without evidence of an infectious cause, determined by conventional cultures or molecular techniques, and 15 from TH caused by *Haemophilus influenzae*. Considering the groups of TH, we enrolled only individuals who underwent a total or partial tonsillectomy due to TH accompanied by snoring, dysphagia, mouth breathing, and/or sleep apnea syndrome, without a history of recurrent tonsillitis [13]. All patients were unrelated individuals and ethnic Greeks, as assessed by questionnaire. A summary of their clinical characteristics is presented in Table 1.

The records of 113 patients with CVID (male/female: 52/61, mean age: 46.2 years, and range: 19–86), derived from the University Hospital of Larissa, Greece, and the University of Freiburg, Germany, for whom the mutational analysis of *TNFRSF13/TACI* has already been published [3, 4], were also retrospectively evaluated for the association of their *TNFRSF13/TACI* status with tonsillar hypertrophy and/or tonsillectomy.

The study was approved by the Institutional Review Boards of the University Hospital of Larissa and the internal ethics review board-approved clinical study protocol (ZERM, number239/99) of the University Hospital of Freiburg, and written informed consent was obtained from each individual or an accompanying relative, in the case of patients where consent was not legally applicable (i.e., children).

### 2.2. Molecular Analyses

Conventional cultures, along with the molecular detection of bacterial DNA and RNA, including the demonstration of *Haemophilus influenzae* carrier state by PCR, were performed on tonsillectomized tissues as described in [13]. 

Extraction of genomic DNA from peripheral blood in patients with sarcoidosis and from tonsillar tissues in patients with TH was carried out by standard protocols [13]. The molecular analysis of *TNFRSF13B/TACI* gene was performed by PCR and sequencing of all 5 exons, including exon-intron boundaries, as described in [4]. 

### 2.3. Statistical Analysis

 Testing for statistical significance of TH with *TNFRSF13B/TACI* mutations in CVID patients was performed using chi-squared (*χ* ^2^) test with Yates' correction. 

## 3. Results 

### 3.1. *TNFRSF13B/TACI* Mutations in Patients with TH and Sarcoidosis

Two patients with TH without infectious cause (incidence 10.5%) and 2 patients with sarcoidosis (incidence 2.8%) displayed *TNFRSF13B/TACI* alterations, all in heterozygous state. No patient with TH caused by *Haemophilus influenzae* carried *TNFRSF13B/TACI* mutations. 

Moreover, we detected several common polymorphic variants within the *TNFRSF13B/TACI* gene, at a similar frequency to that previously described by us in a Greek population with antibody deficiencies [4]. Thus, 22 patients with sarcoidosis were heterozygotes and one homozygous for the common single nucleotide polymorphism (SNP) P251L (rs34562254), and 5 patients were heterozygotes for the other common SNP V220A (rs56063729); among them 2 patients were double heterozygotes for both SNPs previously mentioned. Six patients with TH without infectious causative were heterozygotes for the P251L, while none carried the V220A mutation. Moreover, 6 patients with TH caused by *Haemophilus influenzae* were heterozygotes for the P251L SNP, while one of them also carried the V220A SNP. The allele frequencies of the previously mentioned polymorphisms, the silent mutations 27T (rs8072293), 97P (rs35062843) and 277S (rs11078355), and the intronic polymorphisms g.24625A>C (rs2274892), g.33402T>G (rs11652843), and g.33482T>C (rs11652811) are presented in Table 2. Further analyses indicated no significant differences on the incidence of the previously mentioned SNPs between the subgroups of the analyzed patients (*P* > 0.05 in all cases). 

As mentioned previously, we identified 4 rare variants of *TNFRSF13B/TACI* in the enrolled patients. Considering patients with TH and *TNFRSF13B/TACI* alterations, a 9-year-old boy displayed the mutation I87N (g.24416T>A; rs72553877), along with the common polymorphism P251L, and a 5-year-old girl was heterozygous for the frameshift alteration c.204insA (p.L69TfsX12; rs72553875). The first patient also suffered from neurofibromatosis and displayed TH with mouth breathing and sleep apnea syndrome. Interestingly, he displayed no history of recurrent infections. Further analysis showed normal serum immunoglobulin levels, including IgG subclasses, and sufficient antibody responses to vaccines. Moreover, the mutation was transmitted by his asymptomatic 44-year-old mother, who also exhibited normal serum immunoglobulin levels. Although the I87N mutation has been reported in healthy individuals in a very low frequency (0.001-0.002; http://www.ncbi.nlm.nih.gov/snp/), previous functional studies, analyzing TACI expression and APRIL-binding capacity in EBV-transformed cell lines, have indicated that it is a loss-of-function mutation, since it causes a loss of ligand binding, even in heterozygous state [3]. 

The frameshift alteration c.204insA (p.L69TfsX12; rs72553875) is obviously deleterious, resulting in a premature truncation of the protein [3, 4]. Nevertheless, previous functional studies in single EBV-transformed cell lines carrying a heterozygous c.204insA mutation displayed no significant differences in TACI expression and ligand-binding capacity compared with controls [3]. Our 5-year-old female patient suffered from TH with snoring, dysphagia, and mouth breathing, without a history of recurrent infections. She was an adopted child and her parents refused to be further analyzed (i.e., for immunoglobulin levels).

Considering patients with sarcoidosis, a 42-year-old male was heterozygous for the mutation E36L (g.20820G>A), and a 60-year-old female carried the mutation R202H (g.32987G>A; rs104894649). The E36L mutation is present in SNP database (rs143099385), with an extremely low frequency (<0.0001) in the general population (http://www.ncbi.nlm.nih.gov/snp/). Considering that no functional studies have been carried out for this alteration, we performed bioinformatic analyses using two different software packages, namely, PolyPhen2 (available at: http://genetics.bwh.harvard.edu/pph2/) and SIFT (Sorting Intolerant From Tolerant; available at http://sift.bii.a-star.edu.sg/), to predict its effect on protein function. Both methods indicated that the mutation is probably damaging or deleterious for protein function (PolyPhen2 analysis with a score of 0.995, sensitivity: 0.68, specificity: 0.97 and SIFT analysis with a score <0.01, resp.). Interestingly, the patient did not display any history of recurrent infections or autoimmunity, or family history of immunodeficiency. He exhibited normal serum immunoglobulin levels, including subclasses, and normal levels of anti-PCP (antipneumococcal capsular polysaccharide) and anti-Hbi (anti-*Haemophilus influenzae* type b) antibodies in his blood. The only medical problem was lung sarcoidosis classified by CXR stage II (mediastinal adenopathy with reticulonodular lung infiltrates), which was diagnosed 2 years before enrollment (at age of 40). Lymphoproliferation was also accompanied by skin lesions (granulomatous plaque formations) on the back of the body. The patient received corticosteroids per os for 6 months, with subsequent tapering and achieved a complete remission of the disease. At enrollment into this study, the patient was off treatment. Other family members are not available for mutational analysis until now. 

The second patient was diagnosed with sarcoidosis 12 years ago (CXR stage I with skin involvement) and was a carrier of the R202H mutation. This alteration is also infrequent in the general population (0.001–0.003, http://www.ncbi.nlm.nih.gov/snp/), but it is rather not a loss-of-function mutation, as it behaves like wild-type TACI regarding ligand binding and NFkB transcriptional activity [6]. The patient also suffered from hyperprolactinemia and hypothyroidism, and due to disease deterioration (stage IIIC) she was receiving corticosteroids for 18 months, 8 years ago. Moreover, she displayed a history of recurrent upper respiratory infections (chronic sinusitis) for 10 years before the diagnosis of sarcoidosis, which were treated with oral antibiotics. Interestingly, at enrollment the patient exhibited selective IgG4 deficiency (sIgG4D), with almost undetectable levels of IgG4 in her serum. Molecular analyses of her family members revealed that her 34-year-old son carried also the R202H mutation, yet displaying high IgG4 levels (354 mg/dL, normal range: 8–140), while her 6-year-old grandson (with a mother suffering from CVID) was also heterozygous for the R202H mutation, exhibiting also undetectable IgG4 levels in his serum. Both relatives carrying the R202H mutation have not displayed recurrent infections or signs of autoimmunity, until now. 

### 3.2. Association of Tonsillar Hypertrophy and *TNFRSF13B/TACI* Mutations in CVID Patients

Based on the previously mentioned findings, we further analyzed whether *TNFRSF13B/TACI* alterations predispose to TH in CVID patients. For this purpose, we retrospectively analyzed records of 113 patients with CVID, for whom the mutational status of *TNFRSF13B/TACI* has already been published [3, 4]. Interestingly, 6 out of 14 (42.9%) patients carrying *TNFRSF13B/TACI* defects displayed tonsillar hypertrophy in the past, and 5 of them were also subjected to tonsillectomy. In particular, a patient was homozygous and another one heterozygous in the C104R mutation (rs34557412), one patient was compound heterozygote for both the c.204insA (rs72553875) and the C104R defects, one patient carried the C193X nonsense mutation (rs72553885) and two patients were heterozygotes in A181E defect (rs72553883). On the other hand, 28 out of 99 CVID patients without *TNFRSF13B/TACI* mutations (28.3%) also exhibited tonsillar hypertrophy; although there was a difference in the frequency of TH between two groups, this was not reached to be significant (*P* = 0.422). 

## 4. Discussion

In this study, we investigated if *TNFRSF13B/TACI* alterations, considered either potent susceptibility factors or modifiers of CVID, may also contribute to the development of benign lymphoproliferation. Indeed, our results are in favor of our hypothesis, since the mutations identified in the patients affected by TH without evidence of infectious cause are both deleterious for TACI function and extremely rare in the general population (0.1-0.2%); in addition, the frequency of *TNFRSF13B/TACI* mutations in TH patients (10.5%) was also higher than that expected for the general population (1.2–3.6% in several healthy control groups) [4, 12, 14]. One of the limitations of this study is that the number of patients with TH was rather small; this is partly due to the fact that TH without pathogen cause, established by conventional and molecular techniques, is rather a rare condition. In addition, it cannot be excluded that the individuals carrying the previously mentioned TACI mutations may develop antibody deficiency, like CVID, later in their life. Thus, larger patient cohorts and longitudinal future studies will be needed to draw definite conclusions.

TH is usually a complication of recurrent tonsillitis and also characterized by the isolation of microbial pathogens, either by conventional cultures and/or molecular techniques [15]. However, in some cases, there is no evidence of infectious causative and its pathogenesis is elusive. Considering that, as mentioned previously, TACI abrogation has been related to lymphoproliferation, in both animal and human studies [3, 5, 10, 11], we investigated the role of *TNFRSF13B/TACI* alterations in both TH without evidence of infectious cause and TH caused by *H*. *influenzae*. The latter group of patients was included in our study, since *H. influenzae* is usually part of the tonsillar microbial flora in healthy hosts. Nevertheless, it can be pathogenic when other factors, such as a viral infection, or reduced immune function, create an opportunity [13, 16, 17]. However, no *TNFRSF13B/TACI* mutations were detected in this group of TH patients.

We also identified patients with sarcoidosis carrying heterozygous alterations of *TNFRSF13B/TACI*, but their total frequency (2.8%) was rather low and rather similar to that observed in several healthy control groups [4, 12, 14]. To the best of our knowledge, this is the first study analyzing the prevalence of *TNFRSF13B/TACI* mutations in patients with sarcoidosis. However, Saussine et al. recently reported that BAFF signaling contributes to disease phenotype, since patients with active disease displayed higher BAFF levels, which were also accompanied by hypergammaglobulinemia and higher angiotensin converting enzyme levels, compared to healthy donors and patients with inactive disease [18]. Considering that the number of sarcoidosis patients displaying *TNFRSF13B/TACI* defects in our study was small, the contribution of TACI to disease activity through a defective BAFF signaling is still elusive and remains to be determined. 

Furthermore, we observed that both a patient with sarcoidosis and her healthy grandson carried a heterozygous alteration of *TNFRSF13B/TACI* (R202H) and displayed sIgG4D. Recently, we demonstrated that patients with sIgG4D displayed a higher frequency of *TNFRSF13B/TACI* defects, including R202H, compared to patients with other types of antibody deficiencies [4]. As mentioned previously, *in vitro* studies indicated that the R202H mutation is rather not a loss-of-function mutation [5]; thus, its associations with clinical and phenotypic consequences, including granulomas formation and IgG4 production, need to be further clarified. 

We identified *TNFRSF13B/TACI* mutations only in heterozygous state, raising the question of whether these defects are pathogenic. Previous *in vitro* and animal studies have demonstrated that heterozygous *TNFRSF13B/TACI* mutations alone are sufficient to disturb B-cell function resulting in immunoglobulin production defects and/or lymphoproliferation [5, 6]. On the other hand, heterozygous *TNFRSF13B/TACI* defects have also been identified in healthy individuals, indicating that they rather represent susceptibility factors and/or modifiers of the disease phenotype in patients with CVID [3, 4]. Thus, we suggest that the presence of *TNFRSF13B/TACI* defects might predispose to immune-mediated diseases (ranging from benign lymphoproliferation to overt antibody deficiency) depending on the interaction with other immune gene defects and/or specific environmental factors. 

We observed a higher frequency of TH in CVID patients carrying *TNFRSF13B/TACI* mutations compared to those without mutations; however, this difference was not reached to be significant. Moreover, the contribution of *TNFRSF13B/TACI* mutations in the formation of granulomas in CVID has already been analyzed and no association has been found [3, 4]. However, these findings should be considered with caution, due to the rather low number of CVID patients carrying *TNFRSF13B/TACI* defects, as well as to the fact that the true prevalence of granulomas formation in CVID is rather underestimated, since not all patients with lymphoproliferation and/or hepatic or lung infiltrates are subjected to biopsy. 

 In conclusion, our study further supports the notion that heterozygous alterations of *TNFRSF13B/TACI* alone do not result in overt CVID but may be also found more frequently in distinct clinical phenotypes, including IgG subclass deficiencies, benign lymphoproliferation and, as recently shown, autoimmunity [19] and Good's syndrome [20]. However, taking into account our findings in patients with sarcoidosis and tonsillar hypertrophy, it is obvious that further studies including larger numbers of patients and their relatives may elucidate the precise role of these alterations in the pathogenesis of immune-mediated disorders. 

## Figures and Tables

**Table 1:** Clinical and demographic characteristics of the patients of the study.

Patients with sarcoidosis	
No	71
Sex (male/female)	24/47
Age (mean, range)	49.9, 20–74
Clinical characteristics	
Löfgren's syndrome (*n*, %)	12, 16.9
Ocular involvement (*n*, %)	4, 5,6
Skin involvement (*n*, %)	17, 23.9
Peripheral lymphadenopathy (*n*, %)	3, 4.3
Autoimmune manifestations*	15, 21.1
Chest X-Ray (CXR) staging	
(0) Without mediastinum and lung involvement (*n*, %)	2, 2.8
(I) Mediastinal adenopathy (*n*, %)	13, 18.3
(II) Mediastinal adenopathy + lung involvement (*n*, %)	54, 76.1
(III) Lung involvement only (*n*, %)	2, 2.8
(IV) Lung fibrosis (*n*, %)	0
Corticosteroid treatment (*n*, %)^#^	45, 63.4
Patients with tonsillar hypertrophy without infectious causative	
No	19
Sex (male/female)	11/8
Age (mean, range)	12.1, 3–40
Patients with tonsillar hypertrophy due to *H. influenzae *	
No	15
Sex (male/female)	6/9
Age (mean, range)	10.1, 3–59

*Autoimmune manifestations include the presence of Hashimoto thyroiditis, rheumatoid arthritis, or Sjögren syndrome.

^#^Corticosteroid treatment refers to patients who received either per os or inhaled corticosteroids during the follow-up period.

**Table 2:** Allele frequency (%) of *TNFRSF13B/TACI* polymorphisms in the patients of the study.

Disease	No	Nonsynonymous SNPs		Synonymous SNPs			Intronic SNPs*		
		V220A rs56063729	P251L rs34562254	27T rs8072293	97P rs35062843	277S rs11078355	g.24625A>C rs2274892	g.33402T>G rs11652843	g.33482T>C rs11652811
Sarcoidosis	71	3.52	16.90	71.83	3.52	43.66	48.49	29.58	29.58
TH group 1	19	0	15.79	73.68	2.63	50.00	39.47	26.32	26.32
TH group 2	15	3.33	20.0	80.00	3.33	46.67	50.00	20.00	20.00

SNPs: single nucleotide polymorphisms; TH group 1: tonsillar hypertrophy without infectious causative; TH group 2: tonsillar hypertrophy caused by *H. influenzae.*

*The *TNFRSF13B/TACI* gene numbering is according to GenBank accession number AB22299.
